# Expanding subcutaneous mass on the scalp of an immunocompromised woman

**DOI:** 10.1002/ski2.437

**Published:** 2024-08-07

**Authors:** Ivan Rodriguez, Karen Lam, Simone Montgomery, Shanice McKenzie, Scott Worswick

**Affiliations:** ^1^ Keck School of Medicine University of Southern California Los Angeles California USA; ^2^ Division of Dermatology David Geffen School of Medicine University of California Los Angeles Los Angeles California USA; ^3^ Department of Dermatology Keck School of Medicine University of Southern California Los Angeles California USA

## Abstract

Acute cutaneous presentations in immunocompromised patients demand careful and thorough evaluation. Here, we present the case of a 26‐year‐old female with acute myeloid leukaemia undergoing salvage chemotherapy, who developed an acutely expanding subcutaneous mass starting on the frontotemporal scalp to the angle of the jaw. Histopathologic evaluation was consistent with sclerosing fat necrosis with overlying psoriasiform spongiotic dermatitis. Tissue cultures revealed multi‐drug resistant Pseudomonas aeruginosa. Given these findings, the patient was diagnosed with infectious panniculitis. This case underscores the necessity of comprehensive diagnostic approaches and multidisciplinary collaboration in managing unusual cutaneous presentations in immunocompromised individuals.

## Graphical abstract

This case report describes a 26‐year‐old female with acute myeloid leukaemia undergoing salvage chemotherapy who developed an acutely expanding subcutaneous mass from the frontotemporal scalp to the angle of the jaw. Histopathologic evaluation revealed sclerosing fat necrosis with overlying psoriasiform spongiotic dermatitis, and tissue cultures identified multi‐drug‐resistant Pseudomonas aeruginosa, leading to a diagnosis of infectious panniculitis. The case emphasises the importance of thorough diagnostic approaches and multidisciplinary collaboration in managing uncommon cutaneous presentations in immunocompromised patients.

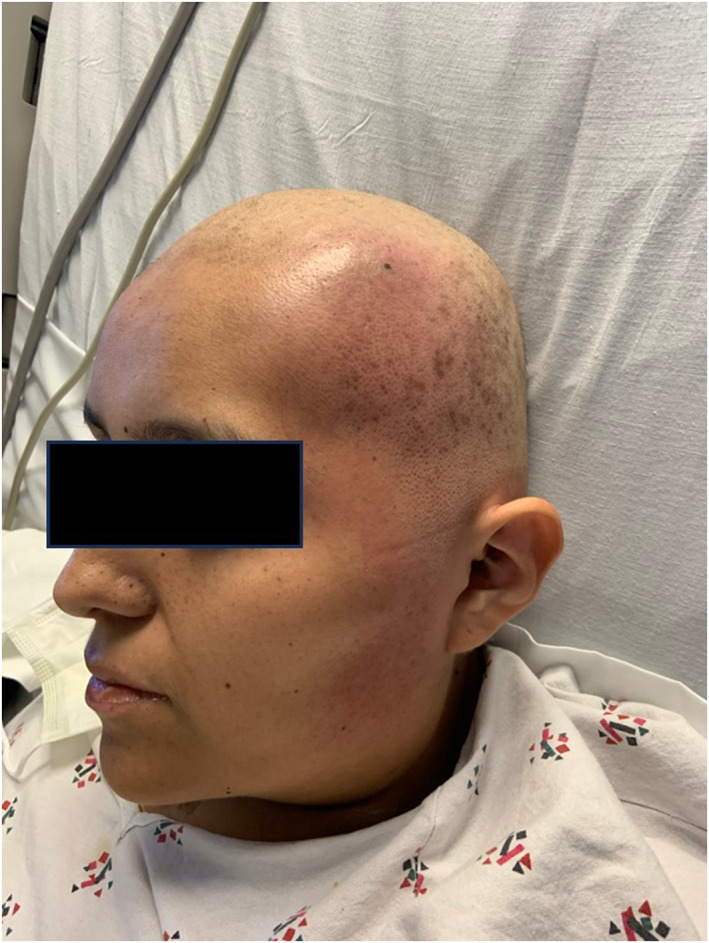

## 1 CASE PRESENTATION

**FIGURE 1:**
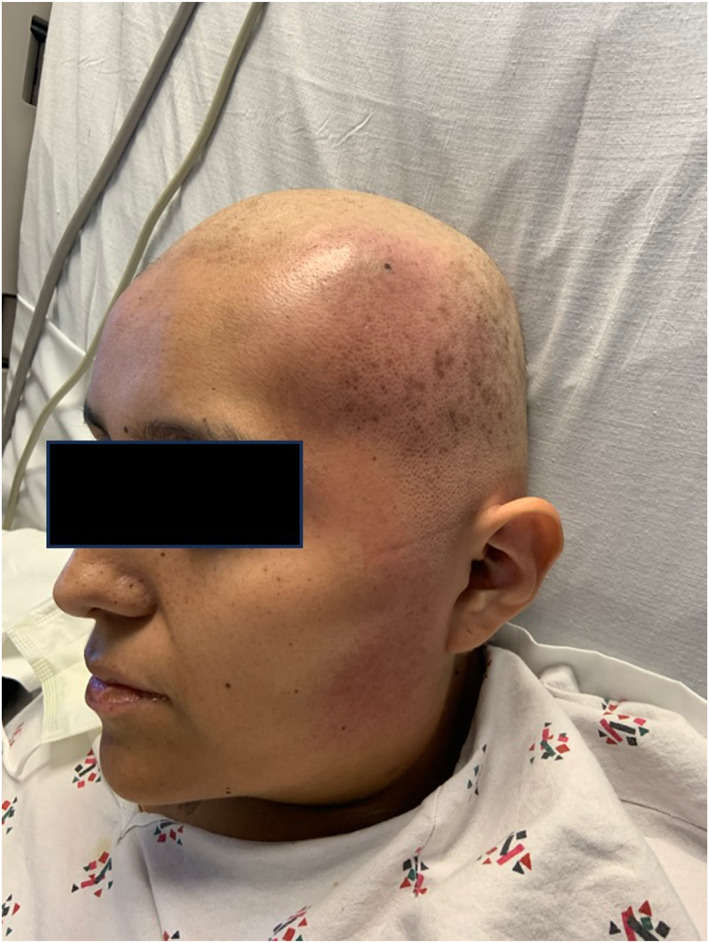
Large, well demarcated plaque with peua d'orange appearance of the left frontotemporal scalp extending to the preauricular areas.

**FIGURE 2:**
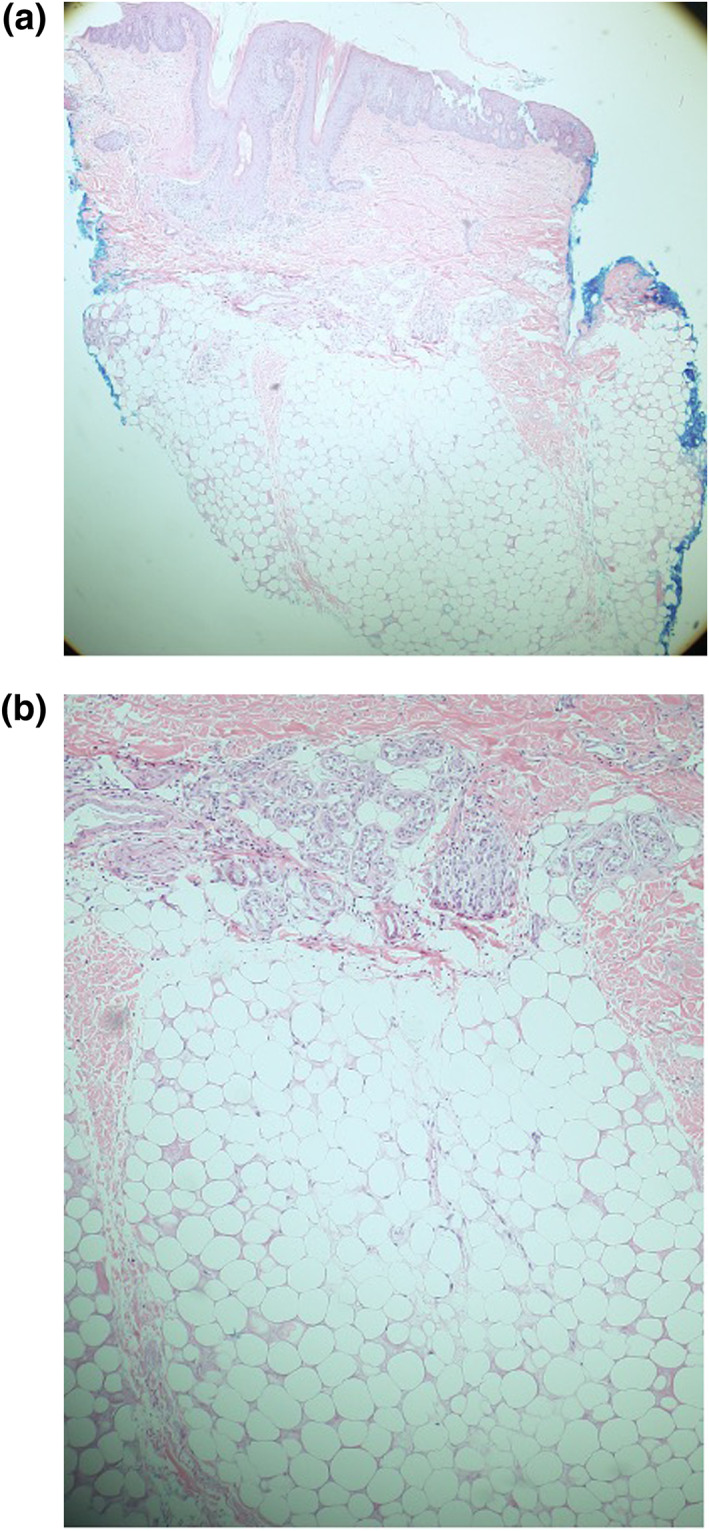
Sclerosing fat necrosis with overlying psoriasiform spongiotic dermatitis (haematoxylin and eosin, ×4) and (haematoxylin and eosin, ×10) respectively.

A previously healthy, 26‐year‐old female with newly diagnosed acute myeloid leukaemia (AML) was admitted for salvage chemotherapy. Her medications included azacytidine and venetoclax for salvage chemotherapy and acyclovir and voriconazole for pancytopenia prophylaxis. Her hospital course was complicated by a peritonsillar abscess, necessitating surgical drainage and broad‐spectrum antibiotics with otolaryngology. The dermatologist was consulted for a rapid‐onset localised swelling with associated pain and erythema on the left temporal scalp. The patient also became febrile to 38.5 C. Her laboratory findings were notable for pancytopenia attributed to chemotherapy. Blood cultures were remarkable for gram‐negative rods and yeast. She was started on meropenem and micafungin for positive blood cultures in the setting of acute febrile neutropenia (FN). Physical examination revealed a well‐demarcated, indurated plaque with a peau d'orange appearance extending from the frontotemporal scalp to the left angle of the jaw (Figure 1). At the centre of the plaque was a focal area of tenderness, erythema, warmth and a brown‐black maculae. A biopsy was performed for tissue cultures and histopathological analysis. Histopathological analysis showed sclerosing fat necrosis with overlying psoriasiform spongiotic dermatitis (Figure 2a,b).

Tissue cultures grew multi‐drug‐resistant (MDR) Pseudomonas aeruginosa, resistant to meropenem. Given the morphologic, histopathologic, and tissue culture findings, the diagnosis of pseudomonal panniculitis was made, and given the bacteriogram results, meropenem was discontinued and ceftolozane/tazobactam was started. Over several days, the patient's erythema, fluctuance and pain improved. Despite demonstrating improvement in her cutaneous symptoms, she began experiencing shortness of breath and haemodynamic instability, suspected to be related to her bacteraemia. The patient was transferred to the ICU and vasopressor support was initiated, but unfortunately, she decompensated rapidly and passed away.

## 2 DISCUSSION

Locoregional pseudomonal panniculitis is a rare manifestation of pseudomonal infection. In previously reported cases, the most common presentation was one or multiple subcutaneous nodules rather than large, indurated plaques as in our patient. It most commonly arises in immunosuppressed patients with bacteraemia as was seen in our patient.^1^ It should be noted that though it is most common, bacteraemia spread is not the only mechanism of spread, with some cases reporting primary cases of pseudomonal cutaneous infection.^2^

While not used in our case, immunostimulant agents such as granulocyte colony‐stimulating factors (G‐CSFs) can be considered prophylactic agents to lower the risk of FN because they can alleviate the pancytopenia caused by chemotherapy. A 2016 systematic review demonstrated a lower risk of FN‐related complications when using pegfilgrastim compared to shorter‐acting G‐CSFs.^3^ In our case, the development of FN was secondary to an infection with MDR pseudomonal infection.

Simply being in the hospital alone is a risk factor for patients. MDR bacteria often develop in hospital settings due to a variety of factors, such as prolonged antibiotic use, lengthy hospital stays and cross‐transmission.^4^ Pseudomonas species are inherently resistant to antibiotics, given their ability to develop biofilms which prevent the penetration of antibiotics to the organisms.^5^ Particularly in nosocomial infections, efflux pumps within the organisms can extrude antibiotics, rendering them ineffective.^5^ MDR pseudomonas aeruginosa infections have mainly been implicated in nosocomial infections and are rarely reported as community‐acquired.^6^ Pseudomonas species are waterborne bacteria and have been shown to colonise hospital plumbing such as sinks, sink traps, faucets, aerators and equipment such as respiratory gear.^5^, ^6^ In a 2020 multicentre retrospective review evaluating pseudomonal bacteraemia in neutropenic cancer patients, 25.4% (309/1217) of patients with bacteraemia were infected with MDR strains.^7^ Preventing transmission from healthcare providers (HCP) to patients is essential, as previous studies have shown 5% of HCPs hands are contaminated with MDR organisms.^8^ Furthermore, recent studies have also demonstrated a risk of MDR on the hands of patients themselves at baseline of entering the hospital.^8^ Especially, when caring for neutropenic patients, effective hand hygiene is critical.

The atypical physical exam findings led to a differential that included Sweet syndrome, leukaemia cutis and toxic erythema of chemotherapy. Sweet syndrome is a neutrophilic dermatosis that classically presents with rapid onset of leucocytosis, associated fevers and tender, erythematous, nodules and/or plaques. It is also known to be associated with haematological malignancies, including AML as in our patient.^9^ However, histopathological analysis shows a dense neutrophilic infiltrate, which was not seen in this case.

Leukaemia cutis involves infiltration of the dermis or subcutaneous tissues with leukaemic cells. It has been reported to present in a cellulitis‐like manner with a similar constellation of symptoms to our patient, including swelling, erythema and tenderness.^10^ In these cases, histopathology would demonstrate infiltration of the dermis or subcutaneous tissues with leukaemic cells. Leukaemia cutis is known to be associated with poor prognosis and should be considered in patients with new skin manifestations with a known underlying haematological malignancy.

Given the onset during salvage chemotherapy and morphology, toxic erythema of chemotherapy (TEC) was also considered. Classically, this presents with erythematous patches or plaques on the hands, feet, or intertriginous areas. TEC can present 2 days to up to 3 weeks after the initiation of chemotherapy, which temporally coincided with our patient's salvage therapy.^11^ It is most often self‐limited and affected areas subsequently desquamate.

This case highlights the importance of considering unusual manifestations of infections, such as pseudomonal panniculitis, particularly for rapid‐onset dermatoses in immunosuppressed patients. Dermatologists should consider pseudomonal panniculitis in patients with positive blood cultures, and dermatoses with morphological features reminiscent of other conditions, including Sweet syndrome, leukaemia cutis, or TEC. Moreover, rapid onset cutaneous findings in the inpatient setting, especially in immunocompromised patients, should carry a low threshold for biopsy and tissue cultures to ensure a thorough diagnostic workup and prompt diagnosis. The challenges encountered in this case emphasise the need for a multidisciplinary approach and a high index of suspicion when dealing with unusual presentations of infections in the context of underlying haematological malignancies or immunosuppression.

## CONFLICT OF INTEREST STATEMENT

None to declare.

## AUTHOR CONTRIBUTIONS

**Ivan Rodriguez**: Data curation (lead); formal analysis (equal); investigation (equal); methodology (equal); writing – original draft (lead). **Karen Lam**: Formal analysis (supporting); writing – review & editing (equal). **Simone Montgomery**: Data curation (equal); formal analysis (equal); writing – review & editing (lead). **Shanice McKenzie**: Formal analysis (equal); writing – review & editing (equal). **Scott Worswick**: Conceptualisation (lead); formal analysis (equal); methodology (equal); supervision (lead); validation (lead); writing – review & editing (equal).

## ETHICS STATEMENT

The University of Southern California Institutional Review Board issued approval HS‐18‐00640. The IRB reviewed this study and determined that it qualifies as exempt 8 under the USC Human Research Protection Programme Flexibility Policy.

## PATIENT CONSENT

The authors obtained informed consent from the patient's next of kin to publish this case report and any accompanying images.

## Data Availability

The data that support the findings of this study are available from the corresponding author upon reasonable request.
